# Unmethylated Insulin as an Adjunctive Marker of Beta Cell Death and Progression to Type 1 Diabetes in Participants at Risk for Diabetes

**DOI:** 10.3390/ijms20163857

**Published:** 2019-08-08

**Authors:** Kimber M. Simmons, Alexandra Fouts, Laura Pyle, Pamela Clark, Fran Dong, Liping Yu, Sahar Usmani-Brown, Peter Gottlieb, Kevan C. Herold, Andrea K. Steck, The Type 1 Diabetes TrialNet Study Group

**Affiliations:** 1 Barbara Davis Center for Childhood Diabetes, University of Colorado Anschutz Medical Campus, 1775 Aurora Ct, MSA140, Bldg 20, Aurora, CO 80045, USA; 2 Department of Pediatrics, University of Colorado Anschutz Medical Campus, Aurora, CO 80045, USA; 3 Department of Immunology, Yale University, New Haven, CT 06520, USA; 4 L2 Diagnostics, New Haven, CT 06511, USA

**Keywords:** type 1 diabetes, biomarkers, prediction, beta cell death

## Abstract

Islet autoantibody (iAb)-positive individuals have a high risk of progression to type 1 diabetes (T1D), although the rate of progression is highly variable and factors involved in the rate of progression are largely unknown. The ratio of unmethylated/methylated insulin DNA levels (unmethylated *INS* ratio) has been shown to be higher in participants at high risk of T1D compared to healthy controls. We aimed to evaluate whether an unmethylated *INS* ratio may be a useful biomarker of beta cell death and rate of progression to T1D. In TrialNet participants who were followed in the Pathway to Prevention Study and progressed to diabetes (*n* = 57, median age of onset 15.3 years), we measured unmethylated *INS* ratio and autoantibodies by electrochemiluminescence (ECL) assays (ECL-IAA, ECL-GADA, and ECL-IA2) and radioimmunoassays (RIA) (mIAA, GADA, IA2A, and ZnT8A) longitudinally for 24 months prior to diagnosis. Linear models were used to test the association between unmethylated *INS* ratio and the age at T1D diagnosis and unmethylated *INS* ratio and iAb over time. Close to diabetes onset, the unmethylated *INS* ratio was associated with mIAA (*p* = 0.003), ECL-IAA (*p* = 0.002), and IA2A (*p* = 0.01) levels, but not with GADA, ECL-GADA, ECL-IA2, or ZnT8A levels. No significant associations were found at baseline (24 months prior to T1D diagnosis). Only mIAA levels were significantly associated with an unmethylated *INS* ratio over time, with a 0.24 change in the ratio for each 0.1 change in mIAA z-score (*p* = 0.02). Adjusting for a baseline unmethylated *INS* ratio, an increased rate of change in unmethylated *INS* ratio from baseline to diabetes onset was associated with a five-year decrease in age at T1D diagnosis (*p* = 0.04).

## 1. Introduction

Type 1 diabetes (T1D) can be identified prior to the onset of clinical symptoms by measuring islet autoantibodies (iAb) to glutamic acid decarboxylase (GAD), insulinoma antigen 2 (IA-2), zinc transporter 8 (ZnT8), and insulin in serum [1]. Prospective cohort studies in Colorado, Finland and Germany that have followed children at high genetic risk for T1D indicate that the development of two or more iAb confers a 70% risk for development of T1D within 10 years and a nearly 100% projected lifetime risk for development of T1D [2]. It is important to note that ZnT8A was not measured systematically in these studies, so rates of iAb positivity may be higher than published if all four major iAb were measured. In the Colorado cohort, 89% of children who developed T1D had two or more iAb [3]. Children who progressed to clinical T1D more rapidly seroconverted at a young age, were female, or had the high-risk HLA DR3/DR4-DQ8 genotype [2,3]. In addition, higher levels of iAb to insulin and IA-2 have been associated with increased risk of progression to T1D [3,4]. 

After iAb are identified and before patients are overtly hyperglycemic, there is progressive destruction of insulin-producing beta cells, which is clinically silent. During this time, iAb can be measured by electrochemiluminescence (ECL) or gold-standard radioassays [5,6,7]. However, because iAb do not directly measure beta cell function, they are not helpful in monitoring the progression of beta cell death leading to clinical T1D. With large-scale iAb screening efforts underway in the United States (Autoimmunity Screening for Kids, Denver, CO) and Europe (Fr1da study, Bavaria), there is a need to develop biomarkers that can better quantify the evolution of beta cell injury and death that leads to clinical T1D. In addition, biomarkers assessing beta cell death would be important to evaluate the effect of interventional drugs on islet cell preservation in T1D prevention trials. 

Circulating unmethylated insulin DNA levels can represent a noninvasive biomarker of beta cell death in T1D [8,9,10,11,12]. In most cells of the body, CpG sites in the *INS* gene are methylated and silenced. Insulin producing beta cells are the only cells in the body with a significant amount of unmethylated *INS* DNA [13,14]. When beta cells are destroyed, as in T1D, beta cell death results in increased unmethylated *INS* in the serum. A study that measured unmethylated *INS* in people at risk of T1D revealed that participants who progress to T1D have higher levels of an unmethylated *INS* ratio compared to healthy controls. The highest unmethylated *INS* ratio was seen in those at highest risk and with a shorter time to T1D diagnosis [15]. Therefore, as a surrogate marker of beta cell death, measuring the unmethylated *INS* ratio could enable evaluation of disease progression during the T1D preclinical period as well as determining efficacy of agents used in prevention trials. 

TrialNet Pathway to Prevention Study screens relatives of T1D patients for iAb and follows these participants with serial longitudinal iAb testing for the development of iAb and type 1 diabetes [16,17]. TrialNet offers close monitoring for iAb-positive participants through HbA1c testing and oral glucose tolerance tests as well as prevention trials [18]. In TrialNet participants who progressed to T1D, we aimed to evaluate whether the unmethylated *INS* ratio is associated with age of T1D onset as well as iAb levels measured by either radioimmunoassay (RIA) or by electrochemiluminecscence assay (ECL).

## 2. Results

### 2.1. Characteristics of TrialNet Participants

TrialNet participants (*n* = 57) were followed in the Pathway to Prevention Study and progressed to diabetes at a median age of 15.3 years (IQR: 10.8–19.0 years). Characteristics of the TrialNet participants at diagnosis are shown in Table 1. The majority of participants were Caucasian, which is representative of the combined demographics at TrialNet Pathway to Prevention enrollment sites. At diagnosis, 85% of participants were positive for one or more iAb by both ECL and RIA measurements with 53% being multiple iAb-positive. Approximately two-thirds of patients were positive for GADA, IA-2A, and ZnT8A by radioimmunoassay (GADA, 70%; IA-2A, 65%; ZnT8A, 65%), while only 24% were positive for mIAA. Using ECL, 42% were positive for GADA, 66% were positive for IA2A, and 25% were positive for mIAA. 

### 2.2. Association of Unmethylated INS Ratio with Age at T1D Diagnosis

**Figure 1:**
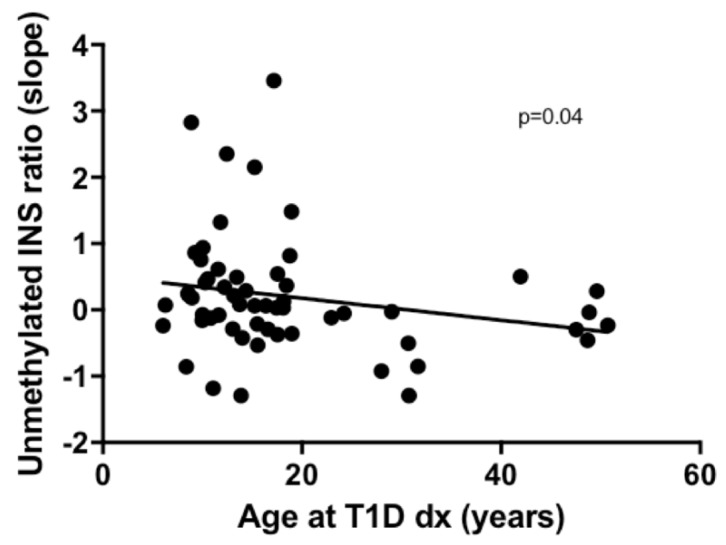
Association between the unmethylated *INS* ratio (ratio of unmethylated/methylated insulin DNA levels) and age at T1D Diagnosis. After adjusting for baseline unmethylated *INS* ratios, an increased rate of change in ratios from baseline to diabetes onset (a slope 10 times steeper) was associated with a five-year decrease in age at T1D diagnosis (*p* = 0.04).

In unadjusted models, the mean, maximum, and slope of the unmethylated *INS* ratio were not associated with age at T1D diagnosis (*p* = N.S.). When adjusting for the value of the unmethylated *INS* ratio at baseline (24 months prior to diagnosis), the slope of the unmethylated *INS* ratio was associated with the age at T1D diagnosis (*p* = 0.04) (Figure 1). An increased rate of change in unmethylated *INS* ratio from baseline to diabetes onset (a slope that was 10 times steeper) was associated with a five-year decrease in age at T1D diagnosis (Figure 1). The larger the change in unmethylated *INS* ratio was over time (slope), the earlier the age at T1D diagnosis. The mean and maximum unmethylated *INS* ratio were not associated with age at T1D diagnosis after adjusting for the unmethylated *INS* ratio at baseline.

### 2.3. Association of Unmethylated INS Ratio with iAb Levels

**Figure 2:**
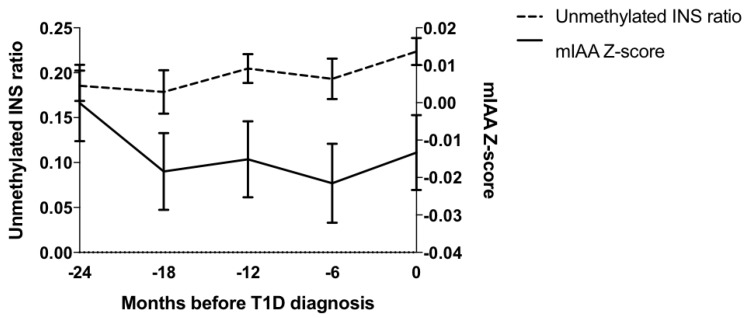
Association of Changes in Unmethylated *INS* Ratio and mIAA Levels (mixed-effects longitudinal models). mIAA levels were significantly associated with unmethylated INS ratios over time, with a 0.24 change in the ratio for each 0.1 change in the mIAA z-score (*p* = 0.02).

Close to diabetes onset (0–2 months prior to diagnosis), unmethylated *INS* ratios were associated with mIAA (*p* = 0.003), IA-2A (*p* = 0.014), and ECL-IAA (*p* = 0.002) levels (Table 2). Unmethylated *INS* ratio was not associated with GADA, ZnT8A, ECL-GADA, or ECL-IA2A levels. There were no significant associations between the level of unmethylated *INS* ratio and iAbs levels at baseline (24 months prior to diagnosis). The unmethylated *INS* ratio was also significantly associated with GADA and ECL-GADA levels at the 18-month visit (*p* = 0.013 and *p* = 0.044, respectively), and with ZnT8A levels (*p* = 0.028) at the six-month visit prior to diagnosis. Mixed-effects longitudinal models were used to examine the association between the changes over time in the unmethylated *INS* ratio and iAb levels. Only mIAA levels were significantly associated with the unmethylated *INS* ratio over time with a 0.24 change in the ratio for each 0.1 change in the mIAA z-score (*p* = 0.02) (Figure 2). 

## 3. Discussion

Although the unmethylated *INS* ratio has been examined in new-onset T1D and populations at high risk for T1D, this is the largest study to our knowledge to look at unmethylated *INS* ratios over time in at-risk participants who progressed to T1D. Importantly, this is the first study to examine the relationship between unmethylated *INS* ratio and age of T1D diagnosis as well as iAb levels over time in at-risk participants who developed T1D.

Islet autoantibodies are not the primary mediators of beta cell injury and death, so although iAb are the main markers of islet autoimmunity, they do not directly measure the degree of beta cell injury. Both mIAA and IA2A levels have been shown to correlate with T1D disease progression. The higher the levels of mIAA and IA2A, the faster a person progresses from iAb positivity to a clinical T1D diagnosis [3,4]. In this study, both mIAA and IA2A were associated with an unmethylated *INS* ratio close to diabetes onset (within 0–2 months prior to diagnosis), but not at baseline (24 months prior to diabetes onset). The fact that these changes and associations are seen close to clinical diagnosis is consistent with several studies that have shown a more acute beta cell loss in the peri-diagnosis period [15,19]. While autoantibodies are important for prediction, other biomarkers are needed to help better define which antibody-positive individuals are at the highest risk for progression to clinical type 1 diabetes. This paper is the first demonstration of a correlation between mIAA and IA2A and beta cell death, although future studies are needed to understand this correlation. The addition of a beta cell death assay, when a patient is positive for these two antibodies, seems to enhance the predictive value of both assays.

Interestingly, mIAA was the only iAb in this study that correlated with unmethylated *INS* ratio over time, with higher mIAA levels associated with a higher unmethylated *INS* ratio. This suggests that mIAA levels may be related to beta cell death and that the presence of mIAA may be important in T1D pathogenesis. Indeed, recent studies have identified T-cells that respond to proinsulin from pancreatic organ donors with recent onset T1D [20]. In addition, effector memory T cells that respond to insulin directly correlate with mIAA levels, suggesting that insulin-specific T cells and B cells interact to promote T1D disease pathogenesis [21].

Age is another predictor of T1D progression as faster progression from iAb positivity to onset of T1D is associated with a younger age at T1D onset [2,3]. In this study, a larger increase in unmethylated *INS* ratio over time was associated with a younger age of T1D onset, suggesting that beta cell death occurs more quickly in younger participants. This is consistent with the more acute clinical presentation of young children, often in diabetic ketoacidosis at diagnosis, as well as with studies confirming lower C-peptide levels at onset and a greater C-peptide loss post-diagnosis in children [22,23]. The addition of a beta cell death assay helps inform that active beta cell death is happening. This assay can help narrow the window/time frame of when the patient is close to developing T1D, although there is significant amount of variation in the assay, especially in younger individuals. The variation that is seen in young individuals could represent the timing of the blood sample draw with a t1/2 of 2.2 h or the fact that insulitis and most likely beta cell killing waxes and wanes with time and location. Therefore, these results need to be validated in a larger study. Nonetheless, this observation could be of importance as it does correlate with the clinical observations of a faster onset and shorter honeymoon period in younger children. 

Additional limitations of this study include visits every six months with measures of beta cell death and iAb possible only every six months. As the average half-life of unmethylated *INS* DNA is relatively short (a couple hours), it is likely we are missing sporadic beta cell killing happening years before diabetes onset until the peri-diagnosis period when beta cell killing is high. C-peptide correlates well with the loss of beta cell mass over time rather than with acute beta cell death, and therefore the unmethylated *INS* ratio is best interpreted in conjunction with c-peptide. Also, samples were collected prospectively in the TrialNet Pathway to Prevention study; however, assays were run on stored frozen samples. Future studies correlating cytokine production of insulin-specific T-cells with mIAA levels and unmethylated *INS* ratio may further clarify the role that mIAA seems to play in beta cell death and T1D pathogenesis. 

In conclusion, this is the first study to show that a higher unmethylated *INS* ratio, a novel marker of active beta cell death, are associated with mIAA levels over time and younger age at time of T1D diagnosis. These findings need to be confirmed in a large prospective cohort of patients at increased risk for T1D.

## 4. Materials and Methods 

### 4.1. Participants

The TrialNet Pathway to Prevention Study screens relatives of type 1 diabetes patients for the presence of iAb and offers close monitoring and/or prevention trials. Autoantibodies to insulin, GAD65, IA-2, and ZnT8 are measured in the Clinical Immunology Laboratory at the Barbara Davis Center, the core immunology lab for iAb testing for the TrialNet study. In TrialNet participants (*n* = 57) who were followed in the Pathway to Prevention Study, progressed to diabetes, and had samples available for analysis, we measured the unmethylated *INS* ratio by droplet digital PCR longitudinally for 24 months prior to diagnosis. All participants included in the analyses had measurements for iAb by radioimmunoassays (RIA) (GADA, mIAA, IA-2A, and ZnT8A) and by electrochemiluminescence (ECL) (ECL-IAA, ECL-GADA, and ECL-IA2). Visits in the two years preceding T1D diagnosis were classified as: Baseline (21 to 27 months prior to diagnosis), 18 months (15 to <21 months prior to diagnosis), 12 months (10 to <15 months prior to diagnosis), 6 months (3 to <9 months prior to diagnosis), and close to diabetes onset (0 to <3 months prior to diagnosis). All participants provided written informed consent, and assent when applicable. All participants involved in this study signed the consent (and assent when applicable) for monitoring in the Pathway to Prevention Study. Written consent was obtained for all adults. Written assent was obtained for participants aged 7–17 years, while their parents/guardians sign the adult consent. The study was approved by the University of Miami Central Institutional Review Board and Colorado Multiple Institutional Review Board, as well as by the TrialNet ancillary study committee. All assays were measured in a blind manner. 

### 4.2. Laboratory Measurements

Beta cell death assay: DNA was isolated from serum using QIAamp DNA Blood Kits, as suggested by the manufacturer (Qiagen, Hilden, Germany), with a modified incubation period of 20 min at 45 °C in the final step. DNA was bisulfite-treated using the EZ DNA Methylation Kit (Zymo Research, Irvine, CA, USA). Insulin DNA with unmethylated and methylated CpG sites was detected by droplet digital PCR as previously described, which includes primer sequences [9,15]. Briefly, each 25-μL volume consisted of Droplet PCR Supermix (Bio-Rad, Hercules, CA, USA), 900 nM of primer, 250 nM of probe, and 5 μL of sample. The mixture and droplet generation oil were loaded onto a droplet generator (Bio-Rad), and the generated droplets were transferred to a 96-well PCR plate. The PCR reaction was run on a thermal cycler with a 10-min activation at 95 °C, 40 repetitions of a two-step amplification protocol (30 s at 94 °C denaturation and 60 s at 58 °C), and a 10-min inactivation step at 98 °C. The DNA content of the droplets was analyzed with a QX100 Droplet Reader (Bio-Rad) and QuantaSoft (Bio-Rad) Analysis software. Discrimination between droplets that contained the target (positives) and those that did not (negatives) was achieved by applying a fluorescence amplitude threshold based on the amplitude read from the negative template control. For each sample, the ratio of unmethylated *INS* DNA to total (i.e., unmethylated + methylated *INS*) was calculated (unmethylated *INS* ratio). The normal level used is an unmethylated *INS* ratio of 0.203, which is the 95th percentile from 165 healthy control subjects. In blinded dilution samples, the R^2^ was 0.97.

#### 4.2.1. Radioassays 

The radioassays for mIAA, GADA, IA-2A, and ZnT8A used in the present study were all performed at the Barbara Davis Center laboratory as the TrialNet reference laboratory and the assay methods were previously published [24,25,26]. In the 2016 Islet Autoantibody Standardization (IASP) Workshop, sensitivities and specificities were respectively 54% and 98% for mIAA, 68% and 100% for GADA, 72% and 100% for IA-2A, and 66% and 100% for ZnT8A.

#### 4.2.2. ECL Assays

ECL assays for both mIAA and GADA have been previously described [6,7,27]. Briefly, serum samples were mixed with both sulfo-tag and biotin-labeled antigen proteins (either proinsulin or GAD65) for overnight incubation at 4 °C. The antigen–antibody complexes with biotin were captured by a streptavidin coated-plate and sulfo-tag gave the signals with electrochemiluminescence. The results were expressed as an index against internal standard positive controls of either insulin or GAD65 monoclonal antibody. The ECL-IA2A assay was adopted from the ECL-GADA assay using the intra-cellular domain of recombinant human IA2 protein. The ECL assay cut-off indexes of 0.006 for mIAA, 0.023 for GADA, or 0.010 for IA2A were set at the 99th percentile over 100 healthy controls and the ECL inter-assay co-efficiencies of variation (CV) were 4.8% (*n* = 20) for mIAA, 8.8% (*n* = 10) for GADA, and 3.6% (*n* = 10) for IA2A, respectively. In the 2016 IASP Workshop, sensitivities and specificities for the ECL assays were 56% and 100% respectively for mIAA, 68% and 99% respectively for GADA, and 72% and 100% respectively for IA2A among patients with newly diagnosed T1D.

### 4.3. Statistical Analyses

Statistical analyses were performed using PRISM (GraphPad Software, Inc., La Jolla, CA, USA) and SAS version 9.4 (SAS Institute, Cary, NC, USA). Proportions were compared using chi-square or Fisher’s exact test. Z-scores for ECL and RIA iAb levels were calculated using the mean and standard deviation for each assay in this sample. The mean, maximum, and slope of the unmethylated *INS* ratio were calculated for each participant. Linear models were used to test the association between the mean, maximum, and slope (change in ratio from baseline to diagnosis) of the unmethylated *INS* ratio and the age at T1D diagnosis, both in unadjusted models and in models adjusted for “baseline” unmethylated insulin gene levels (i.e., the value at month 24 before diagnosis). Similarly, linear models were used to test the association between the unmethylated *INS* ratio and ECL and RIA antibodies at each time point. 

Mixed-effects longitudinal models were used to examine the associations between the changes over time in the unmethylated *INS* ratio and iAb levels. Since our analyses were based on a priori hypotheses, *p*-values were not corrected for multiple testing. *p*-values < 0.05 were considered statistically significant.

## Figures and Tables

**Table 1:** Characteristics of TrialNet Participants at Type 1 Diabetes (T1D) Onset (*n* = 57).

Characteristics	Value
Gender, female	29 (51%)
Age at T1D diagnosis, median (25th, 75th percentile)	15.3 (10.8, 19)
Race/Ethnicity	
White	48 (84%)
Hispanic	4 (7%)
other	5 (9%)
Unmethylated insulin ratio (mean ± SD)	0.22 ± 0.13
Antibody status (RIA)	
Antibody negative	8 (15%)
Single antibody positive	17 (32%)
≥2 antibody positive	29 (53%)
GADA positive	32 (70%)
IA-2A positive	30 (65%)
mIAA positive	13 (24%)
ZnT8A positive	24 (65%)
Antibody status (ECL)	
≥single antibody positive	45 (85%)
GADA positive	22 (42%)
IA-2A positive	18 (34%)
mIAA positive	13 (25%)

Data is n (%) unless otherwise specified. HLA results only available on participants who had at least one positive antibody.

**Table 2:** Association of Unmethylated *INS* Ratio with iAb Levels Close to Onset (within 0–2 months prior to diagnosis).

Variable	Slope	Standard Error	*p*-Value
GADA	−0.021	0.021	0.3337
IA-2A	0.048	0.019	0.0142
mIAA	0.059	0.019	0.003
ZnT8A	0.012	0.024	0.6179
ECL-GADA	0.025	0.017	0.1425
ECL-IA-2A	0.027	0.022	0.2339
ECL-IAA	0.102	0.031	0.002

Electrochemiluminescence (ECL) and radioimmunoassays (RIA) islet autoantibody (iAb) levels converted to SD units away from threshold (z-scores).

## Abbreviations

iAb	Islet Autoantibody
T1D	Type 1 Diabetes
INS	Insulin
ECL	Electrochemiluminescence
RIA	Radioimmunoassay
GAD	glutamic acid decarboxylase
IA-2	insulinoma antigen 2
ZnT8	zinc transporter 8
mIAA	Insulin autoantibody
